# Prospective paired crossover evaluation of potential impact of investigator gender on perceived pain intensity early after acute or scheduled surgery

**DOI:** 10.1186/s13293-023-00508-9

**Published:** 2023-04-25

**Authors:** Anna Sellgren Engskov, Andreas Ydrefors, Karolin el-Jaleb, Jonas Åkeson

**Affiliations:** 1 Department of Clinical Sciences, Anaesthesiology and Intensive Care Medicine, Lund University, Malmö, Sweden; 2 Skåne University Hospital, Carl Bertil Laurells Gata 9, 3rd floor, SE-20502 Malmö, Sweden

**Keywords:** Acute pain, Gender identity, Medical examiners, Pain measurement, Postoperative pain, Postoperative care, Sex, Visual Analog Scale

## Abstract

**Background:**

Postoperative pain is common but often difficult to assess, and there are many potential confounders. Over the last decades, the gender of investigator as well as participant has been found to influence pain perception in both preclinical and clinical studies. However, to our knowledge this has not been studied in various postoperative patients. Objectives of this study were to test the hypotheses that pain intensity levels early after acute or scheduled in- or out-hospital surgery are lower when evaluated by a female investigator, and higher when reported by a female patient.

**Methods:**

In this prospective observational paired crossover study, two investigators of opposite genders independently obtained individually reported pain intensity levels with a visual analogue scale in a mixed cohort of adult postoperative study patients at Skåne University Hospital in Malmö, Sweden.

**Results:**

In total, 245 (129 female) study patients were included and then one female excluded. The study patients rated their intensity of postoperative pain lower when evaluated by a female than by a male investigator (*P* = 0.006), where the male patients constituted the significant difference (*P* < 0.001). Pain intensity levels did not differ between female and male study patients (*P* = 0.210).

**Conclusions:**

Main findings of lower pain intensity reported by males to a female than to a male investigator early after surgery in this paired crossover study in mixed postoperative patients, indicate that potential impact of investigator gender on pain perception should be considered and further evaluated in clinical bedside practice.

*Trial registration* Retrospectively registered in the ClinicalTrials.gov research database on 24th June 2019 with TRN number NCT03968497.

## Highlights

1. Study patients reported significantly lower pain intensity when evaluated by a female than by a male investigator early after surgery.
2. Male patients constituted the statistical significance of this main finding.
3. This finding was independent of pain intensity level, but particularly pronounced at around 3 VAS units, which is the limit where patients usually start calling for pain relief.
4. Postoperative pain intensity levels did not differ significantly between female and male study patients.
5. This paired clinical crossover study shows for the first time, significant impact of investigator gender on pain perception in a mixed cohort of adult postoperative patients after various surgical procedures.

## Background

Postoperative pain has been estimated to affect up to 80% of surgical patients [1, 2], and pain relief after surgery is often insufficient [2–4]. Individual pain perception is multifactorial and influenced by interacting [5] physiological [6] and psychosocial [7–15] factors. The gender of the investigator as well as the patient has been identified as a conceivable bias in pain assessment, which might have an impact in clinical practice.

Preclinical studies have mainly been associated with lower pain sensitivity, reflected as higher pain threshold [16–20] or lower pain intensity [11, 21–26] levels, in study participants evaluated by females. Accordingly, in clinical studies, lower levels of pain intensity have been reported to female investigators by orthopaedic patients with non-surgical pain [27, 28], but no corresponding differences have been found early after cardiac surgery [29] or in emergency care [30–33].

According to three extensive reviews [34–36], the majority of preclinical studies, regardless of pain stimuli characteristics, report lower pain sensitivity in female study participants, also confirmed recently [37]. In contrast, another review found no gender difference in reported intensity levels of various pain modalities [38], which has also been reported by others [16, 19, 25, 39–41].

Contrarily, in a clinical context several studies suggest females to perceive more pain after surgery [42–47], possibly associated with physiological sex [48] and/or psychosocial gender [49] factors including role expectations [25, 50]. However, a recent study [51] reported no gender difference in pain intensity between genders.

To our knowledge, potential impact of gender or sex of healthcare professionals and patients on individually reported intensity levels of pain, has never been evaluated with a paired crossover study design in a postoperative clinical setting managing a diversity of surgical procedures. The term gender, based on social instead of biological characteristics according to the World Health Organization, seems more appropriate than the term sex in this context.

This paired crossover study was designed to test, primarily the hypothesis that being investigated by a female healthcare professional is associated with lower levels of reported pain intensity than by a male, and secondly the hypothesis that female patients report higher levels of pain intensity, regardless of investigator gender, early after various kinds of acute or scheduled in- or out-hospital surgery in a mixed cohort of adult patients.

## Methods

### Study setting

This prospective observational crossover study, approved by the regional Human Research Ethics Review Board in Lund (Approval No. 2018/601) and performed in accordance with the Declaration of Helsinki, was designed to evaluate potential impact of investigator and patient gender on early postoperative pain intensity in a mixed cohort of adult surgical in- and out-hospital patients managed according to local standards of care (SOC) in three post-anaesthesia care units (PACUs) at Skåne University Hospital, Malmö, Sweden.

Pain intensity levels were evaluated twice in each study patient, according to a paired crossover study design, by two investigators of opposite gender. Individual order of evaluation, primarily taking clinical conditions at the PACU into consideration, were continuously coordinated by the investigators to achieve an even overall distribution of their initial evaluations. Both investigators, a 38-year-old female and a 30-year-old male, were last-year medical undergraduate students with normal body mass index (BMI), dressed in regular gender-neutral hospital staff clothing, and fully aware of main purposes of the study.

### Patients

Inclusion criteria were PACU care after scheduled or acute in- or out-hospital surgery, age above 18 years, cognitive and linguistic abilities to participate, and perceived postoperative pain at the time of initial evaluation. Individual physical status was classified according to the American Society of Anesthesiologists (ASA). The study participants were subjected to abdominal, urological, gynaecological, vascular or breast surgery, classified as endoscopic or minimally invasive, laparoscopic or open procedures.

Written informed consent was obtained from each study patient, after oral and written study information—regarding evaluation of pain intensity levels but not potential impact of investigator or patient gender—had been provided shortly before discharge from the PACU.

### Evaluation of pain

Based on oral information read out loudly by the investigator from a predefined protocol, each study participant was asked to assess pain intensity at rest on two occasions early after arrival in the PACU at approximately 15-min interval.

The level of pain intensity was scored between ‘no’ and ‘worst imaginable’ on a horizontally held 100-mm visual analogue scale (VAS) slide ruler, blinded to the patient and subsequently handed over for the investigator to read and record the score with one decimal from the backside.

Pain intensity scores at or above 4.0 VAS units, considered to indicate at least moderate pain, were to be immediately reported to PACU nurses for further clinical measures. During their PACU stay, the study patients were subjected to additional assessments of pain according to local SOC.

### Statistics

A total number of 198 postoperative patients had been calculated to be required to statistically confirm—based on a paired crossover study design—with 95% statistical probability, and 80% statistical power, a difference of 0.2 ± 1.0 VAS units between pain intensity scores obtained by female and male investigators. To account for 20 percent estimated drop-outs, ethical approval was obtained for inclusion of 248 patients.

The Wilcoxon signed rank test was used to compare VAS scores obtained by the female and male investigators, and also to evaluate potential order and carry-over effects by accordingly comparing scores obtained on the first and second study occasions.

Individual differences between VAS scores obtained by the two investigators, and individual mean values of pain intensity, were plotted according to Bland–Altman. Median values of those differences were calculated within defined intervals of individual mean pain intensity and used to assess potential impact of pain intensity on individual differences between VAS scores obtained by the two investigators. The Chi-2 test was used to compare the proportions of lower pain scores obtained by the female and the male investigator, respectively. The Mann–Whitney U-test was used to compare individual mean scores between female and male study patients.

Parametrical data are reported as mean ± standard deviation (SD), and non-parametrical data as median with interquartile range (IQR) in parenthesis.

Levels of probability (*P*) below 0.05 were considered statistically significant.

## Results

### Patients

**Fig. 1:**
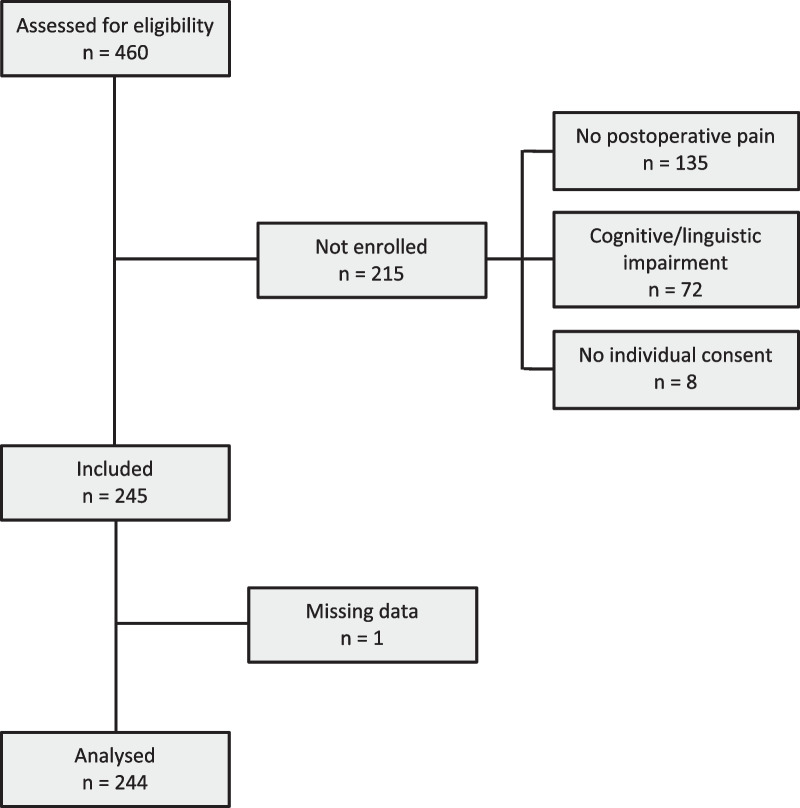
Flowchart of inclusion process. Flow diagram of the inclusion process of study patients

Among 460 patients assessed for eligibility, 214 did not meet inclusion criteria due to absence of postoperative pain, cognitive/linguistic impairment, or lack of individual consent, and one study patient was excluded due to missing data (Fig. 1).

Demographic data are reported in Table 1. In total, data obtained in 244 (128 female) 58 ± 17-year-old study patients, with body mass index (BMI) 26.7 ± 5.2, were analysed. BMI data were missing in one study patient. Of the study patients 47% were at least 65 years, 57% were classified overweight or obese, 29% had severe comorbidity, 65% had abdominal or urological, 29% open, 25% acute, and 13% day, surgery.

**Table 1:** Demographic data of study patients

	Number of patients	Proportion (%)
Gender		
Female	128	52
Male	116	48
Age (years)		
18–29	23	9
30–49	53	22
50–64	53	22
65 +	115	47
Body mass index (kg/m^2^)^a^		
≤ 25.0	102	42
25.1–30.0	94	39
≥ 30.1	47	19
ASA classification		
I	47	19
II	127	52
III	67	28
IV	3	1
Surgical category		
Abdominal	72	30
Urological	86	35
Gynaecological	31	13
Vascular	27	11
Breast	28	11
Surgical technique		
Endoscopic/minimally invasive	97	40
Laparoscopic	76	31
Open	71	29
Surgical planning		
Acute	61	25
Scheduled	183	75
Surgical care		
In-hospital	212	87
Out-hospital	32	13
Total	244	100

Demographic data of the study patients

*ASA* American Society of Anesthesiologists

^a^Data on BMI were not obtained in one study patient

### Evaluation of pain

**Fig. 2:**
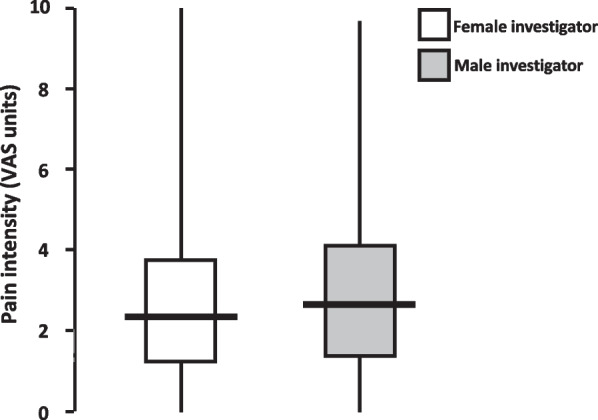
Pain intensity levels obtained by female versus male investigators. Postoperative pain intensity in 244 (128 female) patients, evaluated by both female and male investigators with a crossover study design. Median values are indicated by bold horizontal lines, interquartile ranges by boxes, and ranges by vertical lines. VAS (visual analogue scale)

Of all study patients, 49% were first evaluated by the female investigator. Pain intensity levels (Fig. 2) obtained by the female investigator [median 2.4 (IQR 1.3–3.8) VAS units] were significantly lower (*P* = 0.006) than corresponding levels obtained by the male [2.6 (1.4–4.1) VAS units], as also reflected in lower pain scores obtained in more patients by the female than by the male investigator (139 vs. 93 patients; *P* < 0.001). Compared with the male investigator, the female investigator obtained significantly lower pain intensity levels in males [2.2 (1.2–3.6) vs. 2.5 (1.4–4.0) VAS units; *P* < 0.001], but not in females [2.5 (1.4–3.8) vs. 2.8 (1.4–4.0) VAS units; *P* > 0.300].

**Fig. 3:**
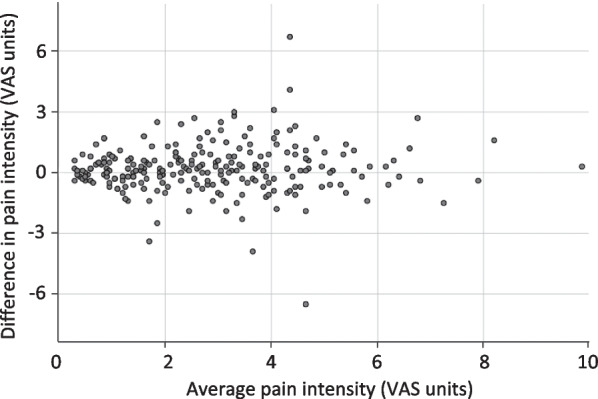
Differences in pain intensity in relation to pain intensity level. Bland–Altman plot of differences between individual pain intensity levels obtained by female and male investigators in 244 (128 female) postoperative patients, and their corresponding average pain intensity levels, evaluated with a prospective crossover study design. VAS (visual analogue scale)

Mild postoperative pain (< 3.5 VAS units) was reported by 66%. The Bland–Altman plot of individual differences between VAS scores obtained by the investigators, and corresponding mean pain intensity (Fig. 3), together with median (IQR) differences calculated within defined intervals of individual mean pain intensity (Table 2), show that patients when evaluated by a female investigator reported lower intensity of postoperative pain regardless of pain level, particularly at around 3 VAS units.

**Table 2:** Differences in pain intensity levels in relation to pain intensity intervals

Average pain intensity (VAS units)	Number of patients	Median (IQR) difference in pain intensity between evaluations by males and females (VAS units)
≤ 1.4	68	0.1 (− 0.2, 0.6)
1.5–2.4	46	0.1 (− 0.4, 0.6)
2.5–3.4	52	0.3 (− 0.4, 0.9)
3.5–4.4	41	0.2 (− 0.6, 1.4)
4.5–5.4	21	0.1 (− 0.6, 0.8)
≥ 5.5	16	0.2 (− 0.4, 1.0)

Differences in pain intensity levels obtained by female and male investigators, within defined intervals of individual mean pain intensity, in 244 (128 female) postoperative patients, evaluated with a prospective crossover study design

*IQR* interquartile range, *VAS* visual analogue scale

Individual mean levels of pain intensity were median 1.9 (IQR 1.2–3.3) VAS units after endoscopic or minimally invasive, 2.9 (1.2–3.3) VAS units after laparoscopic, and 2.9 (1.2–3.3) VAS units after open surgery. None of those median pain intensities were significantly different from each other—endoscopic or minimally invasive vs laparoscopic surgery; *P* > 0.300, endoscopic or minimally invasive vs open surgery; *P* = 0.162, and laparoscopic vs open surgery; *P* > 0.300.

Significantly higher (*P* = 0.015) VAS scores were obtained on the first (2.6 (1.5–4.1) VAS units) than on the second (2.4 (1.2–3.8) VAS units) occasion of individual pain evaluation.

Four study participants (first evaluated by the female investigator) had been given intravenous or oral oxycontin 1 to 7 min before the first pain evaluation, and four participants (three of them first evaluated by the male investigator) had been given intravenous or oral oxycontin eight to eighteen minutes before the second evaluation.

Individual mean levels of pain intensity did not differ significantly (*P* = 0.210) between female and male study patients [median 2.9 (IQR 1.4–3.9) vs. 2.3 (1.3–4.0) VAS units].

## Discussion

Our main findings that (male) patients evaluated by a female investigator report lower intensity of postoperative pain regardless of pain level, have not been reported elsewhere, as far as we know. Although small, we consider these statistically significant differences to be clinically relevant, particularly since they reflect postoperative levels of pain intensity normally calling for analgesic intervention. Moreover, we found no significant difference in pain intensity levels between female and male patients after surgery.

Results obtained in this paired clinical crossover study, carried out in a large cohort of adult PACU patients subjected to various kinds of in- and out-hospital surgery, conform to findings in experimentally induced nociceptive pain in females and males [19] or in males only [17, 24], and also in emergency patients with mild pain [30]. Similar results obtained in patients with foot or ankle pain [27, 28] have possibly been influenced by different medical professions of the investigators and lack of crossover study design.

Similar VAS scores of pain intensity previously obtained by emergency physicians regardless of gender, in young females subjected to pelvic examination [32] as well as in young males subjected to rectal examination [31], most probably reflect unpaired study design and small sample sizes. One smaller unpaired prospective study early after scheduled cardiac surgery [29], and two larger unpaired retrospective studies in prehospital [33] and in-hospital [30] emergency care, have all reported no impact of investigator gender on reported pain intensity, presumably reflecting unpaired study design and low resolution of pain scoring.

The female investigator was found to have significantly influenced pain scoring in males only. Though we also found similar influence in females, we were unable to confirm this statistically. Interestingly, this finding is in line with results of two preclinical pioneer studies within this field [25, 51]. Current knowledge on influence of investigator gender on pain perception has mainly been obtained from preclinical studies in volunteer participants [11, 16–18, 21–26], and as already stated, there are few clinical studies [27, 28, 30–33], and only one in a perioperative context [29]. To our knowledge, this is the first paired clinical crossover study in a large mixed cohort of adult surgical patients, with an even gender distribution and diversity in age, sociocultural background, medical comorbidity, and surgical procedure, which enhances its clinical relevance and general validity.

Considering the short time between distribution of analgesics and evaluation of pain in a handful of patients together with the crossover design, we conclude that early postoperative analgesic interventions have not affected the results overall.

The lack of statistical difference in pain intensity levels between female and male study patients is in agreement with findings in small retrospective [52] and prospective [51] studies in postoperative patients. Contrarily, several studies during the last decade have reported higher pain intensity levels in females after various kinds of surgery [42, 43, 45–47], which might, at least in part, be due to higher pain intensity levels than in our study patients. However, an extensive review based on those results [53] found no consensus regarding associations between patient gender and postoperative pain intensity. Nevertheless, we believe our lack of significant difference in pain intensity between females and males to be reliable, considering the large cohort and mixed surgical procedures followed-up. Moreover, our high proportion of patients with mild postoperative pain [54–56], indicating satisfying pain control, considerably exceeds what has been reported elsewhere after various kinds of scheduled surgery [57]. Our main findings might also be considered to reflect the current general trend towards more laparoscopic and minimally invasive surgical procedures over the last twenty years [58].

The complex relationship between pain perception and investigator gender includes both physiological and psychosocial components, and their interactions with patient and investigator [5]. Lower pain intensity, with no corresponding decrease in heart rate, in male subjects studied by a female [21], has been proposed to result from psychosocial influence on pain perception [2], possibly reflecting perceived traditional gender roles [14, 17, 23, 24]. Nevertheless, the lower scores of pain intensity obtained by the female investigator could, at least in part, have been associated with empathic [9], mainly non-verbal [5], interaction, reported to be more common in females [59, 60] and possibly promoted at the bedside in an intimate PACU setting [8].

Individual- or gender-based verbal influence of the investigators was minimized by predefined study instructions read from a protocol. To avoid interindividual variation, we involved only one study investigator of each gender, whereas previous similar clinical studies in this field [27–33] have included several female and male investigators. Randomization was not possible, since the study patients were not included until considered fully awake shortly before discharge from the PACU after data collection. However, the order of evaluation by female and male investigators was evenly distributed between the study patients. Another limitation of our study design is that individual evaluations by the two investigators could, for practical reasons, not be made simultaneously. We consider approximate 15-min intervals long enough for evaluations not to interfere with each other, and short enough for pain levels not to change considerably. Previous similar paired studies in orthopaedic patients [27, 28] had only 1- to 5-min intervals between individual evaluations, consistently made first by a female nurse and then by a male physician, i.e. without crossover design and by clinical investigators of different medical professions. Influence of order effects on our main findings were compensated for by the paired crossover study design.

Use of VAS scoring—the golden standard for clinical pain assessment—improves the precision in pain scoring and also prevents any impact of auditory memory associated with repeated verbal NRS scoring. On the other hand, since verbal scoring is often used in postoperative routine practice, the clinical relevance of slightly lower VAS scores with a female investigator can be challenged at group level. Nevertheless, these findings might still be relevant to individual patients reporting pain levels where analgesic intervention should be considered.

To reduce potential bias beyond investigator gender, our investigators were equally dressed in hospital clothes, had similar body mass index and represented the same generation and level of education. In contrast to authors referring to gender attributes [17, 24], we did not reinforce potential impact of investigator gender. Psychosocial impact on our study patients was further avoided by carefully blinding them to the main purpose of the study, and by providing oral information according to predefined study protocols.

## Perspectives and significance section

Considering our paired and well-reasoned study design, our results add valuable information in the field of potential gender impact on pain perception. Our main finding is interesting, especially with reference to the majority of female health personnel. This encourages health personnel to have more focus on whether patients are pain evaluated by both female and male staff during the same session, and further on potential impact on their pain assessment. Moreover, hopefully our results will promote future studies regarding investigator gender and pain perception, and subsequently improve evaluation and treatment of acute pain conditions, which might facilitate patient recovery and return to daily life after surgery or trauma.

## Conclusions

This paired clinical crossover study shows for the first time that male patients report significantly lower pain intensity to a female than to a male investigator after various surgical procedures. This should be kept in mind when managing patients with pain in perioperative and emergency care, but further clinical evaluations, with particular reference to underlying factors, are desirable.

## Data Availability

The datasets used and/or analysed during the current study are available from the corresponding author on reasonable request.
